# Elevated circulating myeloid‐derived suppressor cells associated with poor prognosis in B‐cell non‐Hodgkin's lymphoma patients

**DOI:** 10.1002/iid3.616

**Published:** 2022-04-19

**Authors:** Yangyang Wang, Jiyu Wang, Fengfeng Zhu, Huiping Wang, Liuying Yi, Keke Huang, Zhimin Zhai

**Affiliations:** ^1^ Department of Hematology Second Hospital of Anhui Medical University Hefei Anhui China

**Keywords:** B‐cell non‐Hodgkin lymphoma, immunosuppression, myeloid‐derived suppressor cells, prognosis

## Abstract

**Introduction:**

Myeloid‐derived suppressor cells (MDSCs) are a heterogeneous cell population with the ability to suppress immune responses. MDSCs usually cluster in cancer, inflammation, and autoimmune diseases. Although there have been some studies on MDSCs in non‐Hodgkin lymphoma (NHL), the correlation between the peripheral levels of MDSCs in patients with various subtypes of B cell NHL and clinical features and prognosis remains inconclusive. This study aimed at the issue.

**Methods:**

101 patients with B cell NHL and 15 age‐matched healthy controls were included in this study. Flow cytometric detection of monocytic‐MDSCs (M‐MDSCs) and granulocytic‐MDSCs (G‐MDSCs) was done.

**Results:**

In this study, we found that counts of circulating M‐MDSCs and G‐MDSCs were significantly increased in different clinical statuses of B‐NHL patients compared to healthy controls. Similarly, a significant increase in the levels of M‐MDSCs and G‐MDSCs was found among the diverse types of B‐NHL compared with healthy donors. Stratification studies indicated MDSCs expansion was closely associated with disease progression (tumor stage, LDH levels and B syndromes). Moreover, the overall survival time of patients with G‐MDSCs (%) ≥ 98.70% was shorter than patients with G‐MDSCs (%) < 98.70% in newly diagnosed B‐NHL subgroup, meanwhile, there was a significant difference in survival of patients with M‐MDSCs (%) ≥ 7.19% compared to patients with M‐MDSCs (%) < 7.19% in relapsed B‐NHL subgroup.

**Conclusion:**

Our results suggested that M‐MDSCs and G‐MDSCs may be a potential and efficient index to evaluate the prognosis of B‐NHL patients.

## Graphical abstract

In this study, we evaluated the correlation of another immuno‐phenotype myeloid‐derived suppressor cells (MDSCs) (CD14 + CD33 + HLA‐DR^−^^/low^ monocytic‐MDSCs [M‐MDSCs] and CD10‐HLA‐DR^−^^/low^ granulocytic‐MDSCs [G‐MDSCs]) with clinical parameters and disease prognosis of B‐NHL patients. Our results suggested that M‐MDSCs and G‐MDSCs may be a potential and efficient index to evaluate the prognosis of B‐non‐Hodgkin lymphoma (B‐NHL) patients. It may provide a new theory for the pathogenesis of MDSCs in B‐NHL, and more importantly, it may also provide prognostic significance during the clinical treatment of B‐NHL patients.

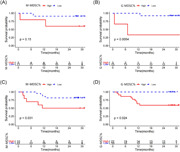

## 1 INTRODUCTION

Non‐Hodgkin lymphoma (NHL) is one of the most common hematological malignancies in the world, and up to 90% of NHLs originate from B cells.^1^ Based on traditional chemotherapy, anti‐CD20 monoclonal antibodies, such as rituximab, brought revolutionary to the clinical treatment of NHL patients.^2^ However, about 35%–40% of NHL patients still face the problem of recurrence after accepting rituximab‐containing therapy,^2^, ^3^ accordingly, outcomes for those patients with threfractory or relapsed diseases have a poor prognosis.^4^ So far, the most used standard clinical tool for evaluating prognosis has been the International Prognostic Index (IPI), but this prognostic scoring system is not able to identify all patients with high‐risk.^5^ This phenomenon means the prognostic scoring system needs further enrichment.

In recent years, the role of myeloid‐derived suppressor cells (MDSCs) has emerged as a clinically applicable biomarker.^6^ As key roles in tumor microenvironment, MDSCs display a potent immune‐suppressive activity towards various immune cells, especially T cells, mainly by the l‐arginine metabolic pathway, therefore immunologically regulate lots of pathological conditions to promote cancer immune evasion.^7^, ^8^ Depending on phenotypic and morphological features, MDSCs can be dissected into two subpopulations: monocytic MDSCs (M‐MDSCs) and granulocytic MDSCs (G‐MDSCs), also known as polymorphonuclear MDSCs (PMN‐MDSCs).^9^ In mice, M‐MDSCs are characterized as clusters of differentiation CD11b + Ly6G + Ly6C+ cells, and G‐MDSCs as CD11b + Ly6G + Ly6Clow.^10^ Regarding human MDSCs, these cells are relatively less well‐characterized due to lacking uniform phenotypic markers. However, they universally express the common myeloid markers CD33 and CD11b, but often lack the maturation marker HLA‐DR.^10^ Previous studies have shown that the increased proportion of MDSCs in many solid cancers could be described as an independent negative prognostic factor.^11^, ^12^, ^13^ Certainly, their prognostic roles in many hematological malignancies (such as Hodgkin's lymphoma, myelodysplastic syndromes, and acute leukemia) have also been extensively explored.^14^, ^15^, ^16^ At present, few studies put focus on comprehensively and systematically analyzing the frequency of MDSCs in B‐NHL patients.

In this study, we evaluated the correlation of another immunophenotype MDSCs (CD14 + CD33 + HLA‐DR^−^^/low^ M‐MDSCs and CD10‐HLA‐DR^−^^/low^ G‐MDSCs) with clinical parameters and disease prognosis of B‐NHL patients. It may provide a new theory for the pathogenesis of MDSCs in B‐NHL, and more importantly, it may also provide prognostic significance during the clinical treatment of B‐NHL patients.

## 2 METHODS

### 2.1 Patients

One hundred and one adult patients diagnosed with B‐cell NHL and 15 healthy adult controls were enrolled in this study from November 2018 to July 2019 in the Second Hospital of Anhui Medical University, including 48 diffuse large B‐cell lymphoma (DLBCL), 10 marginal zone lymphoma (MZL), 12 mantle cell lymphoma (MCL), 12 chronic lymphocytic leukemia (CLL), 14 high‐grade B‐cell lymphoma (HGBL), 4 primary central nervous system lymphoma (PCNSL), and 1 follicular lymphoma (FL). The detailed clinical data of all the enrolled samples are shown in Table 1. All participants with immune or chronic infectious diseases and other types of tumors were excluded from this study. Except for PCNSL and CLL, the rest of the patients were staged based on the Ann Arbor system, and risk stratification was based on the International Prognostic Index (IPI). Peripheral blood samples of all patients were evaluated within 6 h after collection. The research protocol was approved by the Ethics Committee of Anhui Medical University. All the participants obtained written informed consents.

**Table 1:** Characteristics of healthy donors and B‐NHL patients

Groups	Healthy individuals	BNHL	BNHL‐ND	BNHL‐Remission	BNHL‐Relapsed
Average age (range)	51.13 (27–75)	57.55 (23–85)	61.74 (23–82)	54.36 (31–79)	58.35 (23–85)
Gender, number					
Female	8	35	7	22	6
Male	7	66	12	23	28
Lymphoma type, number					
CLL	‐	12	1	4	5
DLBCL	‐	48	10	18	17
HGBL	‐	14	4	10	0
MCL	‐	12	1	3	8
MZL	‐	10	1	7	2
PCNSL	‐	4	0	3	1
FL	‐	1	0	0	1
B syndromes, number	‐				
No	‐	49	11	26	11
Yes	‐	49	8	16	23
LDH levels, number (U/L)	‐				
<120	‐	8	0	5	3
120–250	‐	71	13	30	26
>250	‐	21	6	9	5
IPI score, number	‐				
0	‐	11	1	9	1
1	‐	19	3	10	6
2	‐	20	2	10	7
3	‐	22	4	6	12
4	‐	8	3	0	4
5	‐	6	3	1	4
Ann Arbor stage, number	‐				
I	‐	2	1	0	1
II	‐	13	3	9	1
III	‐	10	2	3	4
IV	‐	54	8	22	22

Abbreviations: B‐NHL, B‐cell non‐Hodgkin's lymphoma; B symptoms, B symptoms refer to systemic symptoms of fever, night sweats, and weight loss which can be associated with B‐NHL; CLL, chronic lymphocytic leukemia; DLBDL, diffuse large B‐cell lymphoma; FL, follicular lymphoma; G‐MDSC, granulocyte MDSC; HDs, healthy donors; HGBL, high‐grade B‐cell lymphoma; IPI, International Prognostic Index; LDH, lactate dehydrogenase; MCL, Mantle‐cell lymphoma; MDSC, myeloid‐derived suppressor cells; M‐MDSC, monocyte MDSC; MZL, marginal zone Lymphoma; ND, newly diagnosed patients with B‐NHL; PCNSL, primary central nervous system lymphoma.

### 2.2 MDSCs analysis

The following monoclonal antibodies were purchased from Beckman Coulter Immunology (Miami): ECD labeled HLA‐DR (clone No. Immu‐375), APC labeled CD14 (clone No. RMO52), PE‐labeled CD33 (clone No. D3HL60.251), FITC labeled CD10 (clone No. ALB1). Peripheral blood mononuclear cells (PBMCs) were extracted by using Ficoll Hypaque (Amersham Biosciences) in all samples. After extraction, samples were processed with phosphate‐buffered saline (PBS) and then 100 ml PBMCs were kept to be incubated with CD antibodies. The flow cytometer (FC500 MPL; Beckman Coulter) was applied for analyzing MDSC cell level, and EXPO 32 Multicomp software was used to collect and analyze the data. All samples were compared to the isotype‐matched antibodies. Then we use forward and lateral scatter histograms to characterize the monocyte population. Next, the expression of HLA‐DR^−/low^ was detected for the monocyte population, and HLA‐DR^−/low^ was gated. We detected the expression of CD14, CD33, and CD10 on HLA‐DR^−/low^ monocytes, respectively, and defined CD14 + CD33 + HLA‐DR^−/low^ cells (M‐MDSCs) and CD10‐HLA‐DR^−/low^ cells (G‐MDSCs).

### 2.3 Statistical analysis

All statistical analysis was performed by using SPSS25 software. Quantitative data were expressed as mean ± standard deviation (SD). Abnormal distribution was expressed as median using nonparametric Mann–Whitney test. To evaluate correlations, Spearman's correlation coefficient was applied. For newly diagnosed (ND) patients, the overall survival (OS) from the diagnosis to death, the last follow‐up or the end of the study was estimated. For relapsed patients, the OS from the relapsed to death, the last follow‐up, or the end of the study was estimated. Kaplan–Meier method was applied for survival curve and univariate analysis. Log‐rank test was applied for evaluating the differences between the comparison of groups. The appropriate cut‐off values of the two MDSCs populations were determined by the maximally selected rank statistics. In ND patients of B‐NHL, the cutoff value of M‐MDSC% was 28.49%, and the cut point of G‐MDSC% was 98.70%. As for relapsed people with B‐NHL, the cutoff value of M‐MDSC% was 7.19%, and the cutoff value of G‐MDSC% was 94.33%. Greater than the respective cutoff value was defined as the high‐count group, and less than or equal to the respective cutoff value was defined as the low‐count group. *p* < .05 was considered statistically significant.

## 3 RESULTS

### 3.1 Increased M‐MDSC% and G‐MDSC% in B‐NHL patients

**Figure 1:**
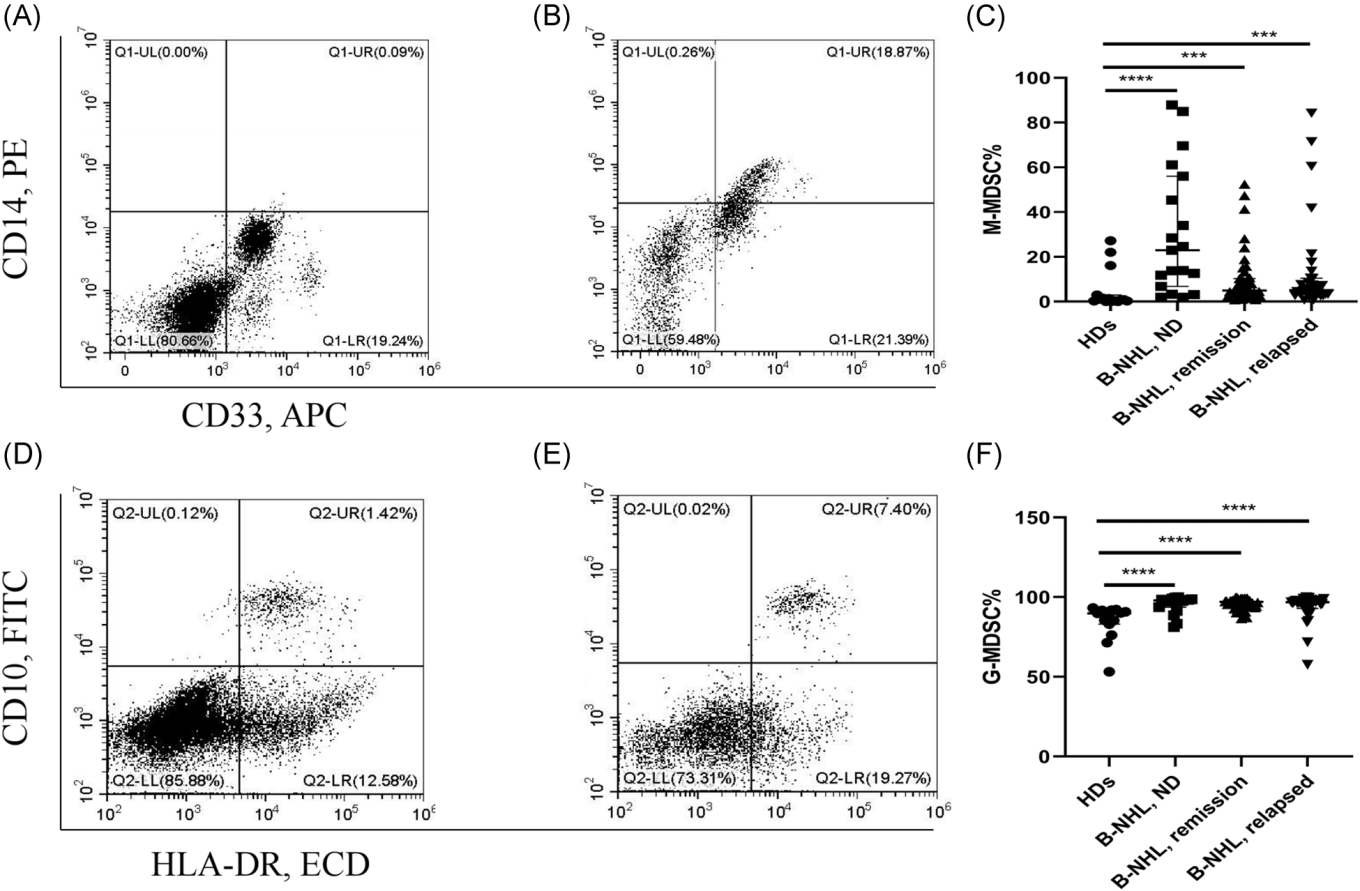
(A) Representative flow cytometry plots of CD14 + CD33 + HLA‐DR^−/low^ (M‐MDSCs) cells in healthy donors. (B) Representative flow cytometry plots of CD14 + CD33 + HLA‐DR^−/low^ (M‐MDSCs) cells in B‐NHL patients. (C) M‐MDSCs in B‐NHL patients of ND, remission and relapsed compared to healthy controls. (D) Representative flow cytometry plots of CD10‐HLA‐DR^−/low^ cells (G‐MDSCs) in healthy donors. (E) Representative flow cytometry plots of CD10‐HLA‐DR^−/low^ cells (G‐MDSCs) in B‐NHL patients. (F) G‐MDSCs in B‐NHL patients of ND, remission and relapsed compared to healthy controls. *p < .05, **p < .01, ***p < .001, **** *p* < .0001, ^ns^ *p* ≥ .05. B‐NHL, B‐cell non‐Hodgkin lymphoma; G‐MDSC, granulocytic‐Myeloid‐derived suppressor cells; M‐MDSC, monocytic‐MDSC; ND, newly diagnosed

Compared with uniform standard of healthy donors (HDs), M‐MDSC% significantly differed between ND subgroup of B‐NHL patients versus normal counterparts (*p* < .0001), remission subgroup of B‐NHL patients vs normal counterparts (*p* < .001) and relapsed subgroup of B‐NHL patients vs normal counterparts (*p* < .001) (Figure 1C). G‐MDSC% in peripheral blood was significantly increased in ND subgroup (*p* < .0001), remission subgroup (*p* < .0001) or relapsed subgroup (*p* < .0001) of B‐NHL patients compared to healthy controls (Figure 1F).

### 3.2 Increased M‐MDSC% and G‐MDSC% in different subtypes of B‐NHL patients

**Figure 2:**
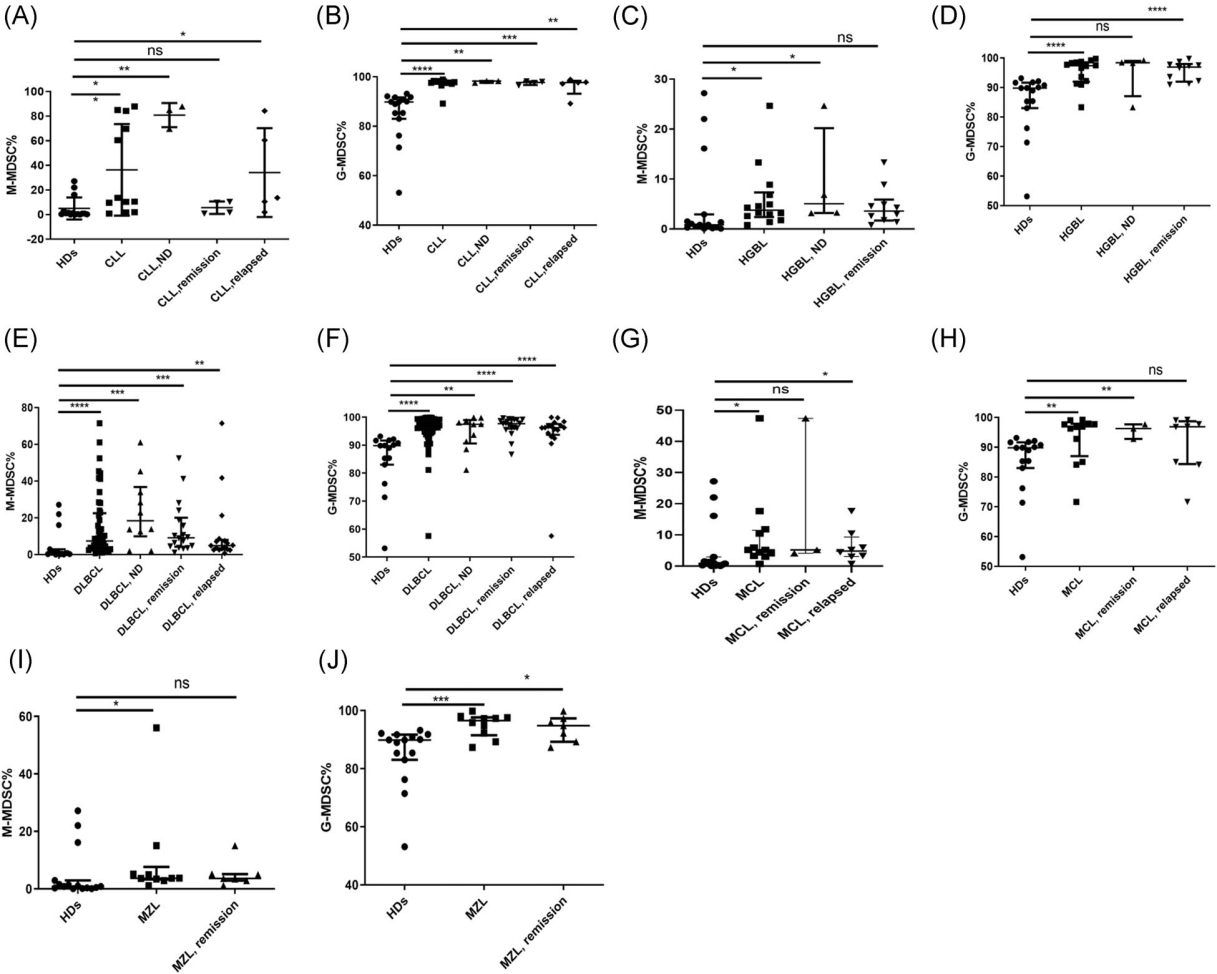
(A, B) M‐MDSCs and G‐MDSCs in CLL patients compared to healthy controls. (C, D) M‐MDSCs and G‐MDSCs in DLBCL patients compared to healthy controls. (E, F) M‐MDSCs and G‐MDSCs in HGBL patients compared to healthy controls. (G, H) M‐MDSCs and G‐MDSCs in MCL patients compared to healthy controls. (I, J) M‐MDSCs and G‐MDSCs in MCL patients compared to healthy controls. CLL, chronic lymphocytic leukemia; DLBDL, diffuse large B‐cell lymphoma; G‐MDSC, granulocyte myeloid‐derived suppressor cells; HGBL, high‐grade B‐cell lymphoma; MCL, Mantle‐cell lymphoma; MDSC, myeloid‐derived suppressor cells; M‐MDSC, monocyte MDSC; MZL, marginal zone lymphoma; PCNSL, primary central nervous system lymphoma. *p < .05, **p < .01, ***p < .001, ****p < .0001, ^ns^ *p* ≥ .05.

A stratified analysis on M‐MDSC% and G‐MDSC% was performed in lymphoma subtypes, including CLL, DLBCL, HGBL, MCL, and MZL. Compared to HDs, M‐MDSCs levels exerted significant difference in the overall CLL group (*p* < .01), CLL‐ND (*p* < .01), and CLL‐relapsed (*p* < .05) subgroups compared to HDs (Figure 2A). Obvious difference was also identified in the analysis of the G‐MDSC% between HDs and total CLL (*p* < .0001), CLL‐ND (*p* < .01), CLL‐remission (*p* < .001), and CLL‐relapsed subgroups (*p* < .01) (Figure 2B).

Compared with HDs, significant difference was observed in the M‐MDSCs levels of either total HGBL patients (*p* < .05) or HGBL‐ND patients (*p* < .05) (Figure 2C). The levels of G‐MDSCs between HDs and the whole HGBL patients exerted significant difference (*p* < .0001), and a similar result was presented between HDs and HGBL‐remission patients (*p* < .0001) (Figure 2D).

As for stratification analysis concerning DLBCL patients, no matter the M‐MDSCs levels or G‐MDSCs levels in any subgroup patients (including total DLBCL, DLBCL‐ND, DLBCL‐remission, and DLBCL‐relapsed subgroups) were significantly meaningful compared to the normal individuals (HDs vs. DLBCL, *p* < .0001; HDs vs. DLBCL‐ND, *p* < .001; HDs vs. DLBCL‐remission, *p* < .001; HDs vs. DLBCL‐relapsed, *p* < .01) (Figure 2E,F).

Then for MCL patients, comparison of the M‐MDSC% between HDs and MCL patients showed a great difference (*p* < .05), and the comparison between HDs and MCL‐relapsed patients showed a similar significant result (Figure 2G). In addition, the levels of G‐MSDC% in the followed comparisons including HDs versus MCL patients (*p* < .01) and HDs versus MCL‐remission patients (*p* < .01) commonly showed significant divergence (Figure 2H).

At last, there apparently existed significant results in the levels of M‐MDSCs between HDs and total MZL patients (*p* < .05) (Figure 2I). However, the G‐MDSCs levels of HDs versus MZL patients (*p* < .001) and HDs versus MZL‐remission patients (*p* < .05) universally showed a significant difference (Figure 2J).

### 3.3 Correlation analysis of clinicopathological factors

**Figure 3:**
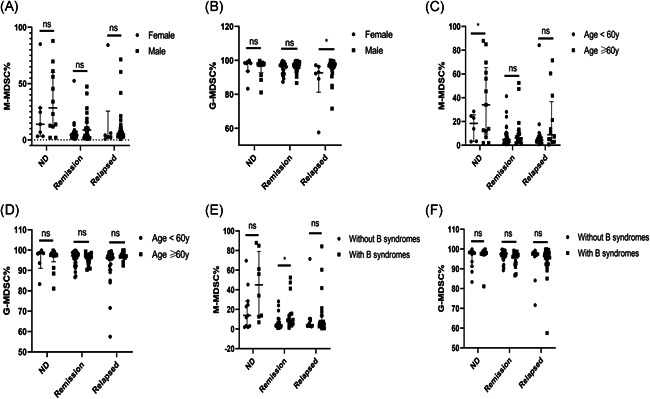
Clinical correlation of the M‐MDSCs and G‐MDSCs levels in different status of B‐NHL patients. (A) No significant difference was detected in M‐MDSC% between female and male groups in ND, remission and relapsed patients. (B) A significant difference was detected in G‐MDSC% between female and male groups in relapsed patients, and there existed no significant difference in ND and remission patients. (C) A significant difference was detected in M‐MDSC% between age <60 y and age ≥60 y groups in ND patients, and no significant difference was found in remission and relapsed patients. (D) No significant difference was detected in G‐MDSC% between age <60 y and age ≥60 y groups in ND, remission, and relapsed patients. (E) A significant difference was detected in M‐MDSCs levels between yes and no groups (Yes, with B syndromes; No, without B syndromes) of remission patients, while no significant difference was detected in ND and relapsed patients. (F) No significant difference was detected in G‐MDSCs between yes and no groups in ND, remission and relapsed patients. Each point represents an individual. *p < .05, ^ns^ *p* ≥ .05. B‐NHL, B‐cell non‐Hodgkin lymphoma; G‐MDSCs, granulocytic‐myeloid‐derived suppressor cells; M‐MDSCs, monocytic‐MDSC; ND, newly diagnosed

**Figure 4:**
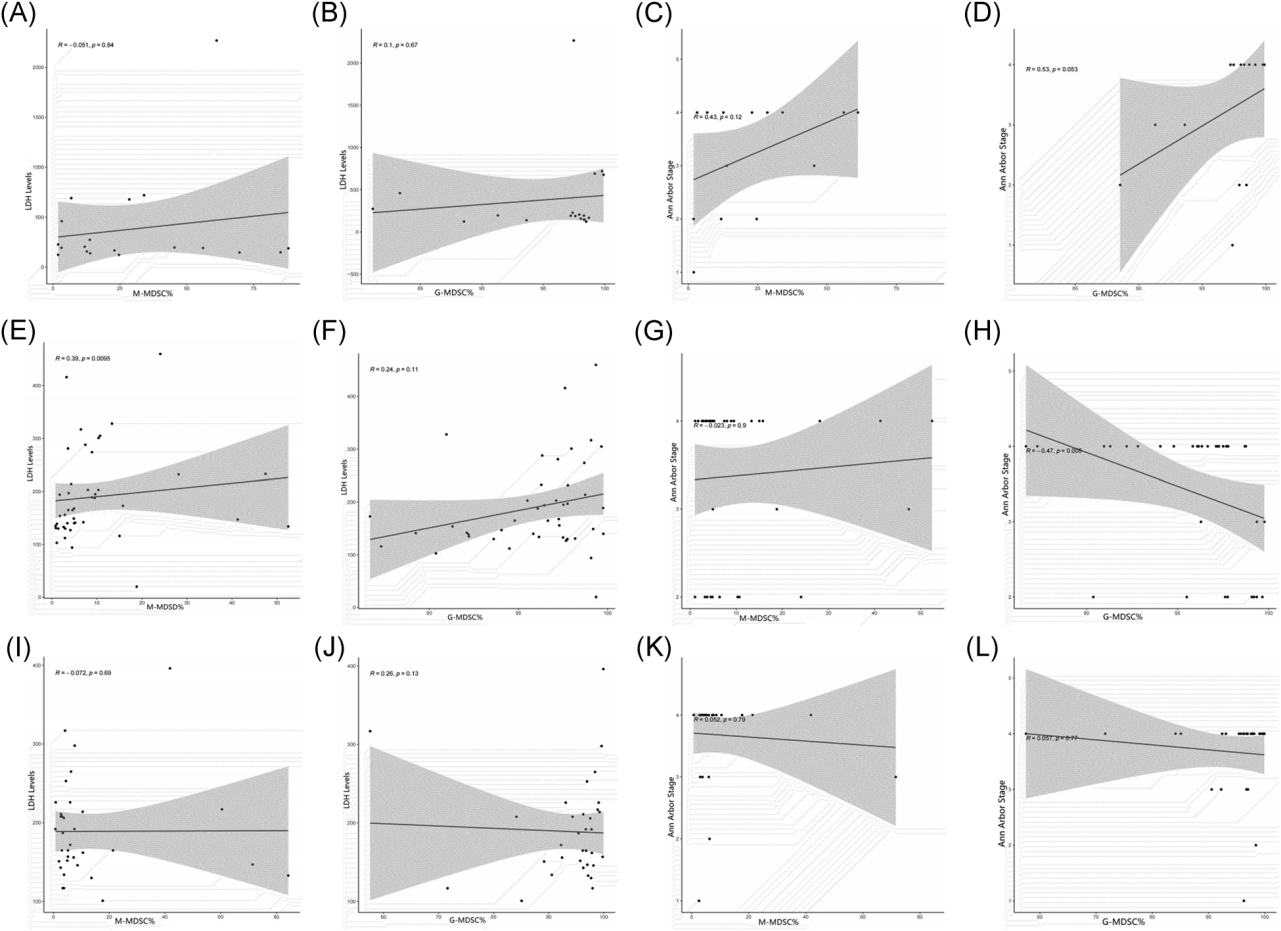
The correlation analysis between MDSCs levels and LDH levels or Ann Arbor Stage in different status of B‐NHL patients. (A–D) No correlation was determined between MDSCs levels and LDH levels or Ann Arbor Stage in ND patients. (E, F) M‐MDSC% was positively correlated with the levels of LDH in remission patients of B‐NHL, while there existed no correlation between G‐MDSC% and LDH levels in remission patients. (G, H) No correlation was determined between MDSCs levels and Ann Arbor Stage in remission patients. (I–L) No correlation was determined between MDSCs levels and LDH levels or Ann Arbor Stage in relapsed patients. Each point represents an individual. The horizontal bar in correlation analysis represents the average. *p < .05, ^ns^ *p* ≥ .05. Each point represents an individual. The horizontal bar in correlation analysis represents the average. *p < .05, ^ns^ *p* ≥ .05. B‐NHL, B‐cell non‐Hodgkin lymphoma; G‐MDSCs, granulocytic‐myeloid‐derived suppressor cells; LDH, lactic dehydrogenase; M‐MDSCs, monocytic‐MDSC; ND, newly diagnosed

In this present study, B‐NHL patients with different clinical status were grouped by types of clinicopathological factors, which included age, gender, B syndromes, lactic dehydrogenase (LDH) levels, Ann Arbor Stage, and IPI scoring system. As for ND patients of B‐NHL, the M‐MDSC% significantly differed between age lower and higher than 60 years subgroups (*p* = .045) (Figure 3C), while no significant difference was found about the G‐MDSC% (Figure 3D). No significant difference was determined in the levels of M‐MDSCs and G‐MDSCs between female patients and male patients (Figure 3A,B). Similarly, we also found no significant results between the patients with B syndromes and without B syndromes (Figure 3E,F). In addition, correlation analysis presented that there existed no association between the MDSCs levels and LDH levels or Ann Arbor Stage (Figure 4A–D), but Figure 4D showed us a trend suggesting the G‐MDSCs levels were positively correlated with the grades of Ann Arbor Stage (*p* = .053).

Considering remission patients of B‐NHL, a significant difference was identified in the levels of M‐MDSCs between the patients with B syndromes and without B syndromes (*p* < .05) (Figure 3E), while no significant association was obtained between the G‐MDSC% and B syndromes (Figure 3F). For age and gender subgroups, there were no significant findings in the stratified analysis of neither the M‐MDSC% nor G‐MDSC% (Figure 3A–D). Additionally, correlation analysis revealed that the M‐MDSCs levels were higher in the high levels of LDH than in the low levels (*r* = .39, *p* = .010) (Figure 4E), while no meaningful results were found about the G‐MDSCs levels (Figure 4F). As for the relationship between MDSCs levels and Ann Arbor Stage, we only obtained a significantly close association in the G‐MDSC% (*r* = −.47, *p* = .005) (Figure 4G,H).

Regarding relapsed patients of B‐NHL, subgroup analysis presented that the G‐MDSC% was significantly associated with gender (*p* < .05) (Figure 3B), while M‐MDSC% was not (Figure 3A). Commonly, there was no significant difference in the analysis about both age and B syndromes subgroups (Figure 3C–F). Significant correlation between either of two MDSC% subgroups and clinical indicators including Ann Arbor Stage and LDH levels was not observed (Figure 4I–L).

**Figure 5:**
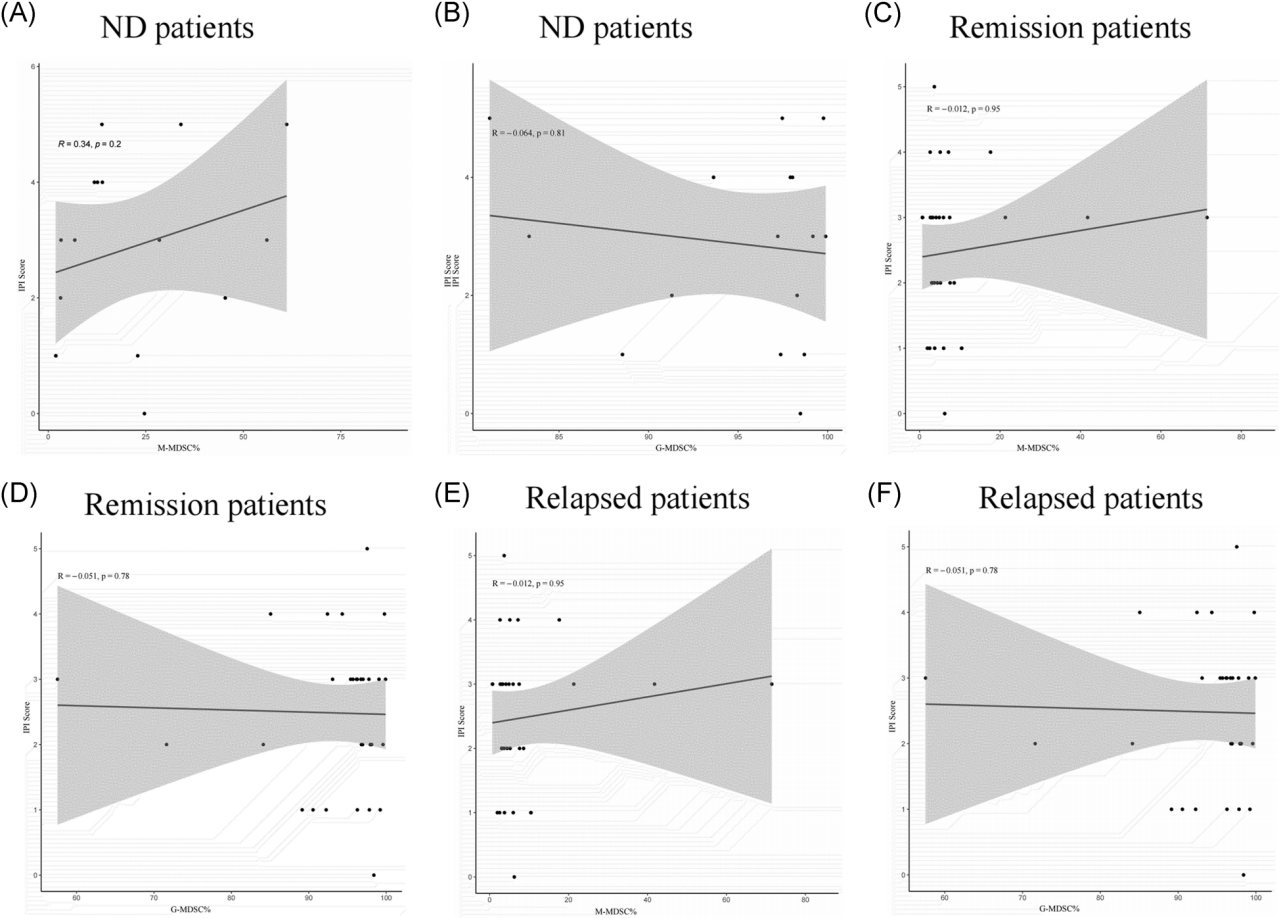
The correlation analysis between MDSCs levels and IPI scoring system in different status of B‐NHL patients. (A, B) No significant difference was detected between levels of M‐MDSC% or G‐MDSC% and IPI scoring system in ND patients. (C, D) No significant difference was detected between levels of M‐MDSC% or G‐MDSC% and IPI scoring system in remission patients. (E, F) No significant difference was detected between levels of M‐MDSC% or G‐MDSC% and IPI scoring system in relapsed patients. B‐NHL, B‐cell non‐Hodgkin lymphoma; G‐MDSCs, granulocytic‐myeloid‐derived suppressor cells; IPI, International Prognostic Index; M‐MDSCs, monocytic‐MDSC; ND, newly diagnosed

Finally, no obvious correlation was determined between the levels of M‐MDSC% or G‐MDSC% and IPI scoring system in ND, remission, and relapsed B‐NHL patients (Figure 5A–F).

### 3.4 The association between M‐MDSC% or G‐MDSC% and survival status of B‐NHL patient

**Figure 6:**
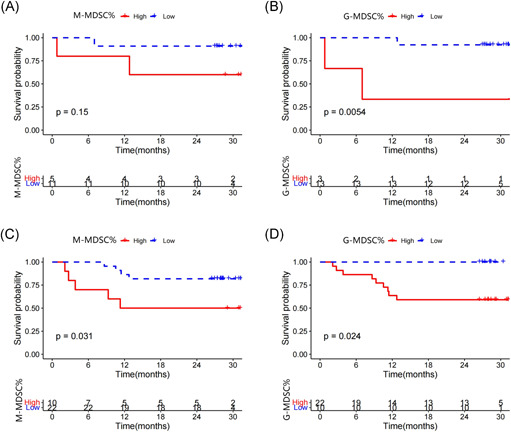
Kaplan–Meier survival curve of overall survival (OS) according to the level of M‐MDSCs and G‐MDSCs. (A) Short OS was shown in high M‐MDSCs groups of B‐NHL ND patients. (B) Short OS was shown in high G‐MDSCs groups of B‐NHL ND patients. (C) Short OS was shown in high M‐MDSCs groups of B‐NHL relapsed patients. (D) Short OS was shown in high G‐MDSCs groups of B‐NHL relapsed patients. B‐NHL, B‐cell non‐Hodgkin lymphoma; G‐MDSCs, granulocytic‐myeloid‐derived suppressor cells; M‐MDSCs, monocytic‐MDSC; ND, newly diagnosed

In our study, the follow‐up time was 0.7–30 months from November 2018 to June 2021. The obviously negative correlation between the OS and the frequency of M‐MDSCs and G‐MDSCs was validated (Figure 6). The ND and relapsed B‐NHL patients were respectively divided into two groups. Regarding the low group (*n* = 11) of ND patients, the levels of M‐MDSC% were defined as less than 28.49%. As for the high group (*n* = 5), the M‐MDSCs levels were greater or equal to 28.49%. No correlation was observed between the ND patients with high M‐MDSCs levels and with low M‐MDSCs levels (Figure 6A). According to G‐MDSC% cutoff value, ND patients of B‐NHL were also divided into two groups. The G‐MDSCs levels of low group (*n* = 13) were less than 98.70%, and the levels of high group (*n* = 3) were greater or equal to 98.70%. Survival analysis equally showed that the poor OS was closely related to high levels of G‐MDSCs (*p* = .002) (Figure 6B). Then for relapsed patients with B‐NHL, the cut points of M‐MDSC% and G‐MDSC% were respectively 7.19% and 94.33%, separately. The Kaplan–Meier analyses showed the OS of relapsed B‐NHL patients with high M‐MDSCs levels or high G‐MDSCs levels was significantly shorter than those with low M‐MDSC levels or low G‐MDSCs levels (*p* < .05) (Figure 6C,D).

Furthermore, other factors possibly associated with clinical outcomes, such as age, gender, B syndromes, LDH levels, IPI score, and Ann Arbor Stage were also be evaluated in this study. For ND patients, the poor OS was related to the existence of B syndromes (*p* = .014), high LDH levels (*p* < .001), and high grades of IPI score (*p* = .027) (Table 2). For relapsed patients, results showed that there existed significant difference in LDH levels between patients with poor prognosis and good prognosis (*p* = .037) (Table 3).

**Table 2:** Survival analysis of prognostic factors in ND patients with B‐NHL

Factor	Survival analysis of ND patients	
	Number	log‐rank *p* value
Age (y)		
≤65	8	0.063
>65	8	
Gender		
Male	10	0.875
Female	6	
B syndromes		
No	10	**0.014**
Yes	6	
LDH levels		
≤678	13	**<0.001**
>678	3	
IPI score		
0–4	11	**0.027**
>4	3	
Ann Arbor stage		
I–III	6	0.081
IV	7	
M‐MDSC%		
≤28.49	11	0.148
>28.49	5	
G‐MDSC%		
≤98.70	11	**0.005**
>98.70	3	

*Note*: MDSC level (high/low) is based on the maximally selected rank statistics. Bold values are statistically significant.

Abbreviations: B‐NHL, B‐cell non‐Hodgkin lymphoma; G‐MDSC, granulocyte myeloid‐derived suppressor cells; IPI, International Prognostic Index; LDH, lactic dehydrogenase; M‐MDSC: monocyte MDSC; ND, newly diagnosed.

**Table 3:** Survival analysis of prognostic factors in relapsed patients with B‐NHL

Factor	Survival analysis of relapsed patients	
	Number	log‐rank *p* value
Age (y)		
≤46	8	NA
>46	8	
Gender		
Male	28	NA
Female	4	
B syndrome		
No	11	NA
Yes	21	
LDH levels		
≤151	9	**0.037**
>151	23	
IPI score		
0‐3	26	0.150
>3	5	
Ann Arbor stage		
I‐III	6	0.105
IV	22	
M‐MDSC%		
≤7.19%	22	**0.031**
>7.19%	10	
G‐MDSC%		
≤94.33%	10	**0.024**
>94.33%	22	

*Note*: MDSC level (high/low) is based on the maximally selected rank statistics. Bold values are statistically significant.

Abbreviations: B‐NHL, B‐cell non‐Hodgkin lymphoma; G‐MDSC, granulocyte myeloid‐derived suppressor cells; IPI, International Prognostic Index; LDH, lactic dehydrogenase; M‐MDSC: monocyte MDSC; ND, newly diagnosed.

## 4 DISCUSSION

MDSCs are a crowd of heterogeneous and immature myeloid progenitors which that originate from tbone marrow and have been defined to be a major regulator in tumorigenesis and tumor progression.^17^, ^18^ Accumulating evidence suggested that MDSCs participated in the immunosuppressive response to many types of cancers, such as breast cancer,^19^ colorectal cancer,^20^ multiple myeloma (MM),^21^ NK/T‐cell lymphoma,^22^ and so on. However, the role of MDSCs in development of B‐NHL has not been fully understood yet. During our work, we explored the potential association between the two immunophenotype MDSCs (CD14 + CD33 + HLA‐DR^−/low^ M‐MDSCs and CD10‐HLA‐DR^−/low^ G‐MDSCs) and clinicopathological characteristics of B‐NHL patients.

First of all, significantly higher levels of M‐MDSCs and G‐MDSCs were detected in ND, remission, and relapsed B‐NHL patients as commonly compared with healthy controls. At the same time, a significant increase in the levels of M‐MDSCs and G‐MDSCs was found among the diverse types of B‐NHL when compared with HDs. Our results were similar to many previous studies, for example, Wu et al. showed that the levels of M‐MDSCs were significantly increased in DLBCL patients compared with healthy controls.^23^ ND lymphoma patients, consisting of 24 patients with HGBCL and 19 patients with classic Hodgkin lymphoma, had more G‐MDSCs than healthy blood donors.^24^ In contrast to previous work, an overall evaluation about different types of B‐NHL tumors was performed in our study and grouped by their condition (ND, remission, and relapsed), indicating that elevated MDSC levels were a common phenomenon in lymphoma. In addition, a large number of patient samples were involved in our analysis, and the results were correspondingly reliable to a high degree.

Then, several classical indicators concerning the clinical status of patients were selected for the purpose of probing the connection between these indicators and the changes of MDSCs levels. Significant differences were shown in both the stratified and correlation analyses, indicating that the frequency of M‐MDSCs and G‐MDSCs were associated with the status of B‐NHL patients, especially in terms of disease progression (tumor stage, LDH level, and B syndrome). This phenomenon suggested that MDSCs expansion could be recognized as a major pathophysiological feature in B‐NHL patients. It is well known that immunosuppression is a major feature of MDSCs.^25^ M‐MDSCs and G‐MDSCs utilize different immunosuppressive mechanisms to suppress the host immune function, including inducing the production of Tregs and mediating the secretion of various cytokines such as arginase‐1 (ARG1), inducible nitric oxide synthase, transforming growth factor‐β (TGF‐β), interleukin 10 (IL‐10), cyclooxygenase 2, indoleamine 2,3‐dioxygenase (IDO) sequestration of cysteine.^26^ A study by Zhang et al. pointed out that the MDSCs levels of NK/T‐cell lymphoma could inhibit the secretion of IFN‐γ but promote the secretion of IL‐10, IL‐17, and TGFβ and Foxp3 expression in T cells.^22^ Besides, Romano et al. found that G‐MDSCs and their function through increased expression of Arg‐1 are related to the progression of MM.^21^ Combined with these previous studies and the results of our work, we could deduce that MDSCs were involved in the development of B‐NHL patients to a certain extent, and were likely to play an important role through the afore‐mentioned immunosuppressive mechanisms. The detailed reasons are summarized as follows: on the one hand, a large number of studies have found that MDSCs were abundant in the bone marrow, blood, and secondary lymphoid organs of tumor patients, and their accumulation was related to clinical stage, metastatic burden, and chemoresistance,^23^, ^27^, ^28^ which were also similar to our findings. On the other hand, the high levels of MDSCs could generate a large number of immuno‐suppressive cytokines, jointly inhibiting the activity of NK cells, CD8^+^ and CD4^+^ T cells, promoting the expansion of Treg, and affecting the antitumor immune response of patients, finally promoting the occurrence and progression of tumors.^26^ The specific mechanism by which they act in B‐NHL patients is the direction for us to further explore in the future.

In addition to immunosuppressive mechanisms of MDSCs, the impact of tumors on the generation and development of MDSCs also deservs our attention. The growth factors produced by tumors are responsible for accelerating the generation of M‐MDSCs and PMN‐MDSCs, meanwhile vigorously recruiting them from the bone marrow to adjacent areas of the tumors, so as to maintain their levels in the blood. Research in patients with non–small‐cell lung cancer expounded that VEGF was a potent chemoattractant for MDSCs,^29^ and another study in mouse has also supported this point.^30^ TNF‐α, another important proinflammatory mediator, was also founded it could increase the quantity and reinforce the suppressive activity of MDSCs.^31^ PGE2 could drive the process of M‐MDSCs differentiating from human hematopoietic stem cells.^32^ It is worth noting that our research group previously proposed that senescent lymphoma cells of relapsed and refractory DLBCL patients might be involved in inducing the generation of immunosuppressive cells such as MDSCs and Treg through secreting a variety of immunosuppressive cytokines (known as senescence‐associated secretory phenotype, SASP), thereby mediating the resistance of tumor apoptosis.^33^ Under the effects of various factors secreted by tumor cells, MDSCs secreted a variety of proproliferative, proinflammatory, and immunosuppressive molecules via activating their own S1PR1‐STAT3, TGF‐β, and other signaling pathways, to make local blood vessels hyperpermeable, to build pre‐metastatic microenvironments, to promote the recruitment, seeding, and expansion of tumor cells, and to provide conditions for the formation of metastases.^34^, ^35^

Based on the complex and close‐knit interaction between MDSCs and tumors, we further explored the relationship between their presence and prognosis in B‐NHL patients. The percentage of the G‐MDSCs population was correlated with poor survivals of ND B‐NHL patients, and the group with a longer OS of relapsed B‐NHL patients had a lower frequency of two MDSCs subgroups. This proved that the levels of M‐MDSCs and G‐MDSCs might be a potential factor affecting the OS in B‐NHL patients. A lot of previous studies usually putted attention on the relationship between MDSCs levels and a specific subtype of lymphoma, and mainly investigated the possible roles of the M‐MDSCs subgroup. Zahran et al. conducted a study aiming at analyzing the frequency of peripheral M‐MDSCs in ND CLL patients and founded that M‐MDSCs were elucidated to be associated with tumor progression and a poor prognosis of CLL patients.^36^ Another research by Wu et al. enrolled 144 ND patients with DLBCL and 30 healthy population and explored the capability of MDSCs identifying patients with a high‐risk of DLBCL.^23^ In conclusion, the combination of M‐MDSC% and IPI scoring system may be useful for predicting the prognosis of DLBCL patients.^37^ Our results were consistent with those articles mentioned above. Regarding the prognosis, we only provided the evaluation results of the KM curve. The results from a cox regression analysis were not presented in our study; it did not exhibit any meaningful conclusion. This situation might be related to the following points. First, B‐NHL patients can be divided in to multiple kinds of subtypes, and there might existed certain confounding factors in the clinical indicators enrolled in our study such as gender, age, disease stage, and so on. Next, there might be collinearity among the factors included in this analysis, which might lead to the effects of some factors be masked. However, considering that the analysis of the KM curve about those factors provided a certainly meaningful result, more samples of patients maybe need to add in our further study to elucidate the prognostic value of MDSCs in B‐NHL patients.

To conclude, counts of circulating M‐MDSCs and G‐MDSCs were significantly increased in B‐NHL patients. High frequency of circulating M‐MDSCs and G‐MDSCs closely related to tumor progression and poor prognosis of B‐NHL patients. Elevated circulating M‐MDSCs and G‐MDSCs could be defined as effective indicators of poor prognosis in B‐NHL patients. It would be beneficial to prognostic evaluation of patients to monitor these cell populations during the treatment. Controlling the expansion and accumulation of MDSCs represents promising novel approaches in cancer therapy.

## CONFLICTS OF INTEREST

The authors declare no conflicts of interest.

## ETHICS STATEMENT

This study was approved by the Institutional Review Board Institutional of the Second Hospital of Anhui Medical University.

## Data Availability

The data used to support the findings of this study are available from the corresponding author upon request.
